# Extraction of phenolic compounds from extra virgin olive oil by a natural deep eutectic solvent: Data on UV absorption of the extracts

**DOI:** 10.1016/j.dib.2016.05.076

**Published:** 2016-06-03

**Authors:** Vito Michele Paradiso, Antonia Clemente, Carmine Summo, Antonella Pasqualone, Francesco Caponio

**Affiliations:** University of Bari, Department of Soil, Plant and Food Sciences, Via Amendola 165/a, I-70126 Bari, Italy

**Keywords:** Natural deep eutectic solvents, Extra virgin olive oil, Phenolic compounds, UV spectrophotometry

## Abstract

This data article refers to the paper “Towards green analysis of virgin olive oil phenolic compounds: extraction by a natural deep eutectic solvent and direct spectrophotometric detection” [1]. A deep eutectic solvent (DES) based on lactic acid and glucose was used as green solvent for phenolic compounds. Eight standard phenolic compounds were solubilized in the DES. Then, a set of extra virgin olive oil (EVOO) samples (*n*=65) were submitted to liquid–liquid extraction by the DES. The standard solutions and the extracts were analyzed by UV spectrophotometry. This article reports the spectral data of both the standard solutions and the 65 extracts, as well as the total phenolic content of the corresponding oils, assessed by the Folin–Ciocalteu assay.

Specifications Table

**Table:** 

Subject area	*Agricultural and biological sciences*
More specific subject area	*Green chemistry*
	*Analysis of phenolic compounds in vegetable oils*
Type of data	*Figure, Excel files*
How data was acquired	*UV spectra: liquid–liquid extraction (or direct solubilisation for standard phenolic compounds) followed by UV absorption analysis (Agilent Cary 60 spectrophotometer, Agilent Technologies, Santa Clara, USA).*
	*Phenolic compounds content: liquid-liquid extraction followed by Folin-Ciocalteu assay*
Data format	*Pre-processed*
Experimental factors	*Oil samples were directly submitted to liquid-liquid extraction*
Experimental features	*A 6:1:6 lactic acid:glucose:water DES was prepared. Standard phenolic compounds were directly solubilized in DES. Each oil sample was submitted to both water-methanol and DES extraction in duplicate. Water/methanol extracts were submitted to the Folin-Ciocalteu assay for determination of phenolic compounds content. DES extracts were submitted to direct UV absorption analysis*
Data source location	*Bari, Italy*
Data accessibility	*Data is provided with this article*

Value of the data

• This data is available for spectrum processing
• UV spectra of some standard phenolic compounds solubilized in the DES under investigation are available for spectra comparisons and evaluation of spectroscopic properties of phenolic compounds
• Multivariate analysis can be carried out on the data to obtain models to relate the phenolic compounds content (assessed by Folin–Ciocalteu assay on water–methanol extracts) with spectral properties of DES extracts

## 1 Data

Supplementary material S1 reports the UV absorption spectra in the range 252–360 nm of 8 standard phenolic compounds – belonging to different chemical classes: benzoic acid derivatives (hydroxybenzoic acid, protocathecuic acid, vanillic acid), cinnamic acid derivatives (*p*-coumaric acid, caffeic acid), phenyl-ethyl alcohols (tyrosol), flavonoids (apigenin), lignans (pinoresinol) – after solubilization in the DES based on lactic acid and glucose.

Supplementary material S2 reports the phenolic compounds content, expressed in mg gallic acid/kg oil, of the 65 EVOO samples.

**Fig. 1:**
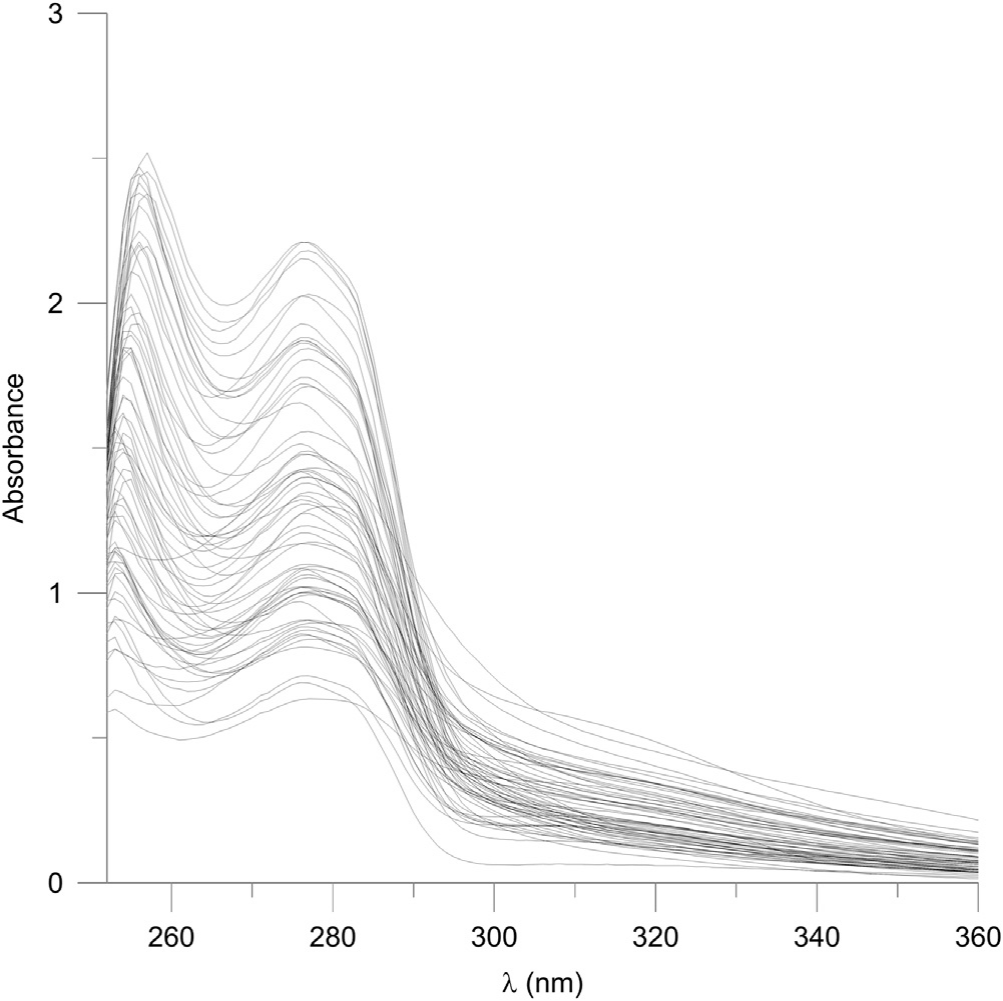
UV spectra of the DES extracts of the 65 EVOO samples.

Supplementary material S3 and Fig. 1 report the UV absorption spectra in the range 252–360 nm of the DES extracts of the 65 EVOO samples. Mean spectra of the two independent extractions after sample weight normalization are reported.

## 2 Experimental design, materials and methods

### 2.1 Reagents and samples

Glucose (≥99.5%), lactic acid (90%), methanol (≥99.8%), Folin–Ciocalteu reagent, and phenolic standards were purchased from Sigma-Aldrich (Sigma-Aldrich Co. LLC, St. Louis, USA). Hexane (≥95.0%) was purchased from Carlo Erba reagents (Carlo Erba reagents, Milan, Italy). Sodium carbonate was purchased from J.T. Baker (Avantor Performance Materials, Center Valley, USA). Sixty-five EVOO samples were obtained from producers and local sellers.

### 2.2 DES preparation

The DES was obtained by mixing lactic acid, glucose and water (6:1:6 M ratio, according to Dai et al. [2], with a slight modification to reduce solvent viscosity), by means of magnetic stirrer at 50 °C for about 90 min, until obtaining a clear solution.

### 2.3 Preparation of standard solutions

The DES solutions (100 mg L^−1^) of the following standards were prepared: hydroxybenzoic acid, protocathecuic acid, vanillic acid, tyrosol, *p*-coumaric acid, caffeic acid, apigenin, pinoresinol.

### 2.4 Extraction and determination of phenolic compounds

Phenolic compounds of the EVOO samples were extracted and determined according to Caponio et al. [3]. Extraction was carried out on 1 g of oil by adding 1 mL of hexane and 5 mL of methanol/water (70:30 v/v). After vortexing for 10 min and centrifuging at 6000 rpm for 10 min at 4 °C (Beckman Coulter, Fullerton, California, USA), the hydroalcoholic phase was recovered, centrifuged again at 9000 rpm for 5 min at 4 °C and filtered through nylon filters (pore size 0.45 μm, Sigma-Aldrich, Milan, Italy). Then, 100 µL of extract were mixed with 100 µL of Folin–Ciocalteu reagent and, after 4 min, with 800 µL of a 5% (w/v) solution of sodium carbonate. The mixture was then heated in a water bath at 40 °C for 20 min and the total phenol content was determined at 750 nm by an Agilent Cary 60 spectrophotometer (Agilent Technologies, Santa Clara, USA). The total phenolic content was expressed as gallic acid equivalents (mg/kg).

### 2.5 Extraction with DES

One g of oil was added with 1 mL of hexane and 5 mL of DES. After intense agitation with vortex, a centrifugation was performed for 10 min at 6000 rpm. The supernatant was subjected to further centrifugation for 5 min at 9000 rpm. The supernatant was then filtered through a 0.45 µm nylon filter. Two independent extractions were carried out for each oils sample.

### 2.6 Acquisition of UV spectra of DES extracts

The DES extracts were analyzed in the wavelength range 240–400 nm by means of an Agilent Cary 60 spectrophotometer (Agilent Technologies, Santa Clara, USA). The acquisition parameters were the following: 1 cm optical path, 2 nm slit, 60 nm min^−1^ scan rate. Pure DES was used for background correction. Mean spectra of the two independent extractions after sample weight normalization were obtained.

## Appendix A Supplementary material

Fig. 1. UV spectra of the DES extracts of the 65 EVOO samples.

## Footnotes

Appendix A Supplementary data associated with this article can be found in the online version at: doi:10.1016/j.dib.2016.05.076.
