# Supplementary material for: Extraction of phenolic compounds from extra virgin olive oil by a natural deep eutectic solvent: Data on UV absorption of the extracts
Source: Data Brief. 2016 Jun 3;8:553–6. doi: 10.1016/j.dib.2016.05.076 (PMC4961719; doi:10.1016/j.dib.2016.05.076)
Supplement: Supplementary file 1 — Supplementary material [file mmc1.pdf]

Bari, May 20th 2016

Manuscript No.: DIB-D-16-00381
Title: Extraction of phenolic compounds from extra virgin olive oil by a natural deep eutectic solvent: data on UV absorption of the extracts
Journal Title: Data in Brief
Corresponding Author: Dr. Vito Michele Paradiso

Conflict of interest form

The authors whose names are listed immediately below certify that they have NO affiliations with or involvement in any organization or entity with any financial interest (such as honoraria; educational grants; participation in speakers' bureaus; membership, employment, consultancies, stock ownership, or other equity interest; and expert testimony or patent-licensing arrangements), or non-financial interest (such as personal or professional relationships, affiliations, knowledge or beliefs) in the subject matter or materials discussed in this manuscript.

Vito Michele Paradiso

Antonia Clemente

Carmine Summo

Antonella Pasqualone

Francesco Caponio

This statement is signed by all the authors to indicate agreement that the above information is true and correct

Vito Michele Paradiso — 20th May 2016

Antonia Clemente — 20th May 2016

Carmine Summo — 20th May 2016

Antonella Pasqualone — 20th May 2016

Francesco Caponio — 20th May 2016
