# Supplementary figures and images for: Extraction of phenolic compounds from extra virgin olive oil by a natural deep eutectic solvent: Data on UV absorption of the extracts
Source: Data Brief. 2016 Jun 3;8:553–6. doi: 10.1016/j.dib.2016.05.076 (PMC4961719; doi:10.1016/j.dib.2016.05.076)

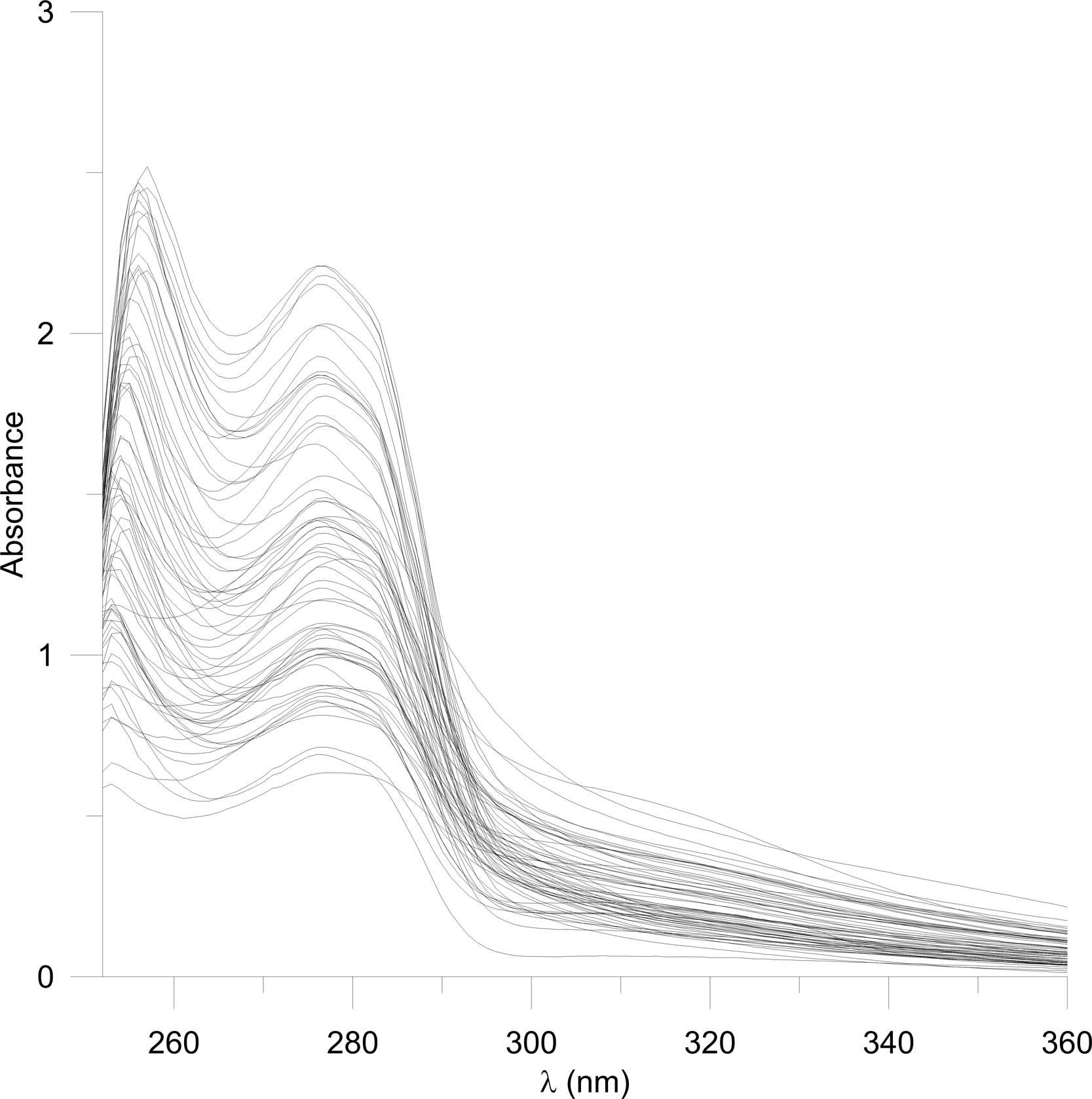

Supplement: Supplementary file 2 — Fig. 1. UV spectra of the DES extracts of the 65 EVOO samples. [file mmc2.zip › mmc2.tif]
